# Supplementary material for: Crystal Engineering of Ionic Cocrystals Sustained by Azolium···Azole Heterosynthons
Source: Pharmaceutics. 2022 Oct 28;14(11):2321. doi: 10.3390/pharmaceutics14112321 (PMC9696709; doi:10.3390/pharmaceutics14112321)
Supplement: Supplementary file 1 [file pharmaceutics-14-02321-s001.zip › pharmaceutics-1941155-supplementary.pdf]

Supplementary Materials

Crystal Engineering of Ionic Cocrystals Sustained by Azolium···Azo+le Heterosynthons

Maryam Rahmani, Vijith Kumar, Julia Bruno-Colmenarez and Michael J. Zaworotko *

Department of Chemical Sciences and Bernal Institute, University of Limerick, Limerick V94 T9PX, Ireland
* Correspondence: xtal@ul.ie

Table of contents……………………………………………………………………………………………1, 2

Section S1: Synthesis of ionic cocrystals reported herein ……………………………………………………3

General procedures………... ………………………………………………………………………………. 3

Table S1. Details on the cocrystallization processes. ……………………………………………………4

Table S2. Chemical structures of new salts synthesized in approach II. ………………………………………5

Specific procedures………... ……………………………………………………………………………… 6

1.1. DMIHBA ……………………………………………………………………………………………6

1.2. BEPTEX……………………………………………………………………………………………..6

1.3. MPIHBA·2H_2O ……………………………………………………………………………………...6

1.4. MPINIA and MPINIA·2H_2O …………………………………………………………………………..6

1.5. MPITSA……………………………………………………………………………………………...6

1.6. MPITFA……………………………………………………………………………………………...6

1.7. IMBHBA……………………………………………………………………………………………7

1.8. NPIHBA……………………………………………………………………………………………7

1.9. NPIHCA……………………………………………………………………………………………7

1.10. NPINIA……………………………………………………………………………………………7

1.11. NPITFA……………………………………………………………………………………………7

1.12. MPTHBA…………………………………………………………………………………………7

1.13. MPTHCA and MPTHCA.2H_2O………………………………………………………………….8

1.14. MPTTFA……………………………………………………………………………………………8

1.15. MPIBMPT…………………………………………………………………………………………8

Section S2: Comparison of experimental and calculated PXRD patterns…………………………………9

Figure S1. Experimental and calculated PXRD patterns of DMIHBA. ………………………………………9

Figure S2. Experimental and calculated PXRD patterns of BEPTEX. …………………………………………9

Figure S3. Experimental and calculated PXRD patterns of MPIHBA.2H_2O. ……………………………………10

Figure S4. Experimental and calculated PXRD patterns of MPINIA and MPINIA.2H_2O. …………………10

Figure S5. Experimental and calculated PXRD patterns of MPITSA. …………………………………………11

Figure S6. Experimental and calculated PXRD patterns of MPITFA. …………………………………………11

Figure S7. Experimental and calculated PXRD patterns of IMBHBA. …………………………………………12

Figure S8. Experimental and calculated PXRD patterns of NPIHBA ………………………………………12

Figure S9. Experimental and calculated PXRD patterns of NPIHCA. …………………………………………13

Figure S10. Experimental and calculated PXRD patterns of NPINIA. ………………………………………13

Figure S11. Experimental and calculated PXRD patterns of NPITFA. ………………………………………14

Figure S12. Experimental and calculated PXRD patterns of MPTHBA. ………………………………………14

Figure S13. Experimental and calculated PXRD patterns of MPTHCA. ………………………………………15

Figure S14. Experimental and calculated PXRD patterns of MPTHCA.2H_2O. …………………………………15

Figure S15. Experimental and calculated PXRD patterns of MPTTFA. ……………………………………………16

Section S3: Cambridge Structural Database (CSD) analysis……………………………………………………. 17

Scheme S1. CSD statistics for supramolecular synthons in structures containing azole rings. …………………. 18
Table S3. CSD statistics for supramolecular synthons found in structures containing azole rings. ……………. 19

Section S4. Crystal structure analysis. ………………………………………………………………………….20

Figure S16. Illustration of isostructural ICCs DMIHBA (a) and BEPTEX (b) along c axis. ……………………..……20
Figure S17. Graph-set analysis of water molecules in MPIHBA.2H_2O…………………………………………………20
Figure S18. The chain arrangement of nitrate anions and water molecules in MPINIA.2H_2O. ………………..……20
Figure S19. Graph-set analysis of water molecules in MPTHCA.2H_2O. ……………………………………….....…21

Section S5. Hydrogen bond analysis. ……………………………………………………………………...…...22

Figure S20. CSD statistics for azole-ring structures. ………………………………………………………..…... 22

Section S1: Synthesis of ionic cocrystals reported herein

General procedures. All of the acids and solvents were obtained commercially and utilized without additional purification. The chemicals 1,2-dimethylimidazole and 4-(1H-imidazole-1-yl) benzaldehyde were purchased from Sigma Aldrich (+98 % GC), as were 1-(4-methoxyphenyl)-1H-imidazole (+98 % GC) and 1-(4-methoxyphenyl)-1,2,4-triazole (+99 % GC) from Alfa Aesar, and 1-(4-nitrophenyl)-1H-imidazole was purchased from AmBeed (+98% GC). All chemicals were used in the experiments exactly as they were received.

Cocrystallization via Solvent Evaporation.

Synthesis of ICCs was carried out by dissolving the reactants in appropriate solvents and warming at 50 °C on a hot plate and subsequent slow evaporation at room temperature (See Table S1). For a typical crystallization, in a 10 mL glass vial, 0.1 mmol of model compounds and stoichiometric amounts of the acids listed in Table S1 were dissolved in 2 mL of the appropriate solvents. The solution was left undisturbed to evaporate under ambient conditions. The crystals of 1-16 were removed from their mother liquors before complete evaporation of the solvent had occurred.

Cocrystallization via Solvent-Drop Grinding.

Stoichiometric amounts of model coformer and related salt (Table S2) were ground using a mortar and pestle for approximately 10 minutes after the addition of methanol (MeOH) or acetonitrile (MeCN) (10 μL of solvent for 50 mg of starting ingredients). The resulting powders were analysed by X-ray powder diffraction.

Table S1. Details on the cocrystallization processes.

	ICC	Composition	Solvent	Method	ΔpK$_a$
1	**DMIHBA**	2DMI+1HBA	MeOH	SDG	15.84
2	**BEPTEX**	2DMI+ 1HCA	MeCN/MeOH (2:1)	SE	13.44
3	**MPIHBA·2H$_2$O**	2MPI+ 1HBA+ 2H$_2$O	EtOH	SDG	14.76
4	**MPINIA**	2MPI+ 1NIA+ 2H$_2$O	MeCN/MeOH (2:1)	SE, SDG	7.44
5	**MPINIA·2H$_2$O**	MPI+ 0.5NIA	MeCN/MeOH (2:1)	SE, SDG	7.44
6	**MPITSA**	2MPI+ 1TSA	MeCN	SE, SDG	21.06
7	**MPITFA**	2MPI + 1TFA	MeCN	SE, SDG	7.40
8	**IMBHBA**	2IMB + 1HBA	MeCN/MeOH (2:1)	SE, SDG	13.74
9	**NPIHBA**	2NPI + 1HBA	MeCN/MeOH (2:1)	SE, SDG	13.46
10	**NPIHCA**	2NPI + 1HCA	MeCN/MeOH (2:1)	SE	11.04
11	**NPINIA**	1NPI + 0.5NIA	MeCN/MeOH (2:1)	SE, SDG	6.17
12	**NPITFA**	2NPI + 1TFA	MeCN/MeOH (2:1)	SE, SDG	19.73
13	**MPTHBA**	2MPT + 1HBA	MeCN	SE	10.26
14	**MPTHCA**	2MPT + 1HCA	MeCN/MeOH (2:1)	SE, SDG	7.86
15	**MPTHCA·2H$_2$O**	2MPT + 1HCA	EtOH/H$_2$O (5:1)	SE, SDG	7.86
16	**MPTTFA**	2MPT + 1TFA	MeCN	SE, SDG	16.56
17	**MPIBMPT**	1MPI + 1HBA + 1MPT	MeOH	SDG	-

Table S2. Chemical structures of new salts synthesized in approach II.

	HBA	HCA	NIA	TSA	TFA
**DMI**	Salt1 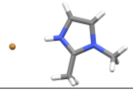	Salt2 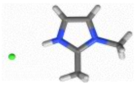	-	Salt3 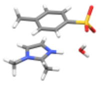	-
**MPI**	Salt4 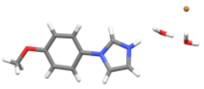	Salt5 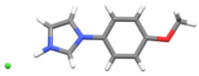	Salt6 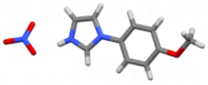	Salt7 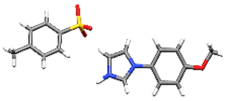	Salt8 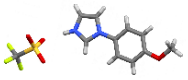
**IMB**	Salt9 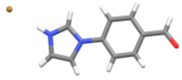	Salt10 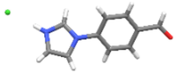	-	-	Salt11 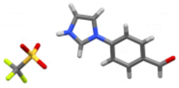
**NPI**	Salt12 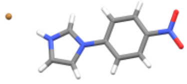	Salt13 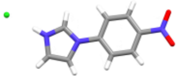	Salt14 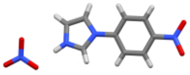	Salt15 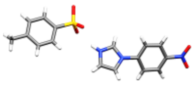	Salt16 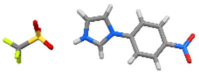
**MPT**	Salt17 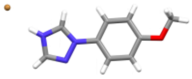	Salt18 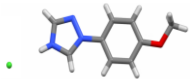	Salt19 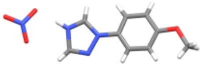	Salt20 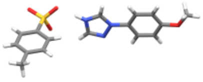	Salt 21 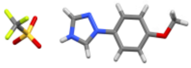

Specific procedures.

1.1 DMIHBA

1,2-dimethylimidazolium bromide, salt1, (0.176 g, 1 mmol) and DMI (0.096, 1 mmol) were ground with the addition of 10 μL of MeOH. By recrystallizing the resulting powder in MeOH after 8 days, **DMIHBA**'s yellowish crystals were formed (Figure S1).

1.2 BEPTEX

DMI (0.096 g, 1 mmol) and 1 M dilute hydrochloric acid (0.5 mL, 0.5 mmol) were dissolved in 2 mL of a 2:1 MeCN/ MeOH solution by warming at 50 °C. After 3 weeks, colorless blocks of **BEPTEX**, were harvested by slow evaporation of solvent (Figure S2).

1.3 MPIHBA·2H_2O

256.09 mg (1 mmol) of 1-(4-methoxyphenyl)-1H-imidazolium bromide, salt4, and 174.20 mg (1 mmol) of MPI were ground with the addition of 10 μL of EtOH. The obtained powder was used for recrystallization in 2 mL of EtOH under ambient conditions. Colorless needle-like single crystals of **MPIHBA·2H_2O** were obtained within 9 days (Figure S3).

1.4 MPINIA and MPINIA·2H_2O

MPI (174.20 mg, 1 mmol) and dilute nitric acid (0.5 mmol, 500 μL) were dissolved in 2 mL of a 2:1 MeCN/MeOH solution and gently evaporated at room temperature. This sample contained crystals with two different morphologies. Colorless needle crystals of **MPINIA**, and block shape crystals of **MPINIA·2H_2O**. In the SDG method, 255.2 mg (1 mmol) of 1-(4-methoxyphenyl)-1H-imidazolium nitrate, salt6, and 174.20 mg (1 mmol) of MPI were ground with the addition of 10 μL of MeCN (as an impurity) (Figure S4).

1.5 MPITSA

MPI (261.10 mg, 1.5 mmol)) and dilute p-toluenesulfonic acid (0.5 mmol, 500 μL) were added to 2 mL of MeCN and slowly evaporated under ambient conditions. Colorless block crystals of MPITSA were obtained within 13 days and were used for single-crystal X-ray crystallography. In the SDG method, 346.4 mg (1 mmol) of 1-(4-methoxyphenyl)-1H-imidazolium tosylate (salt7) and 174.20 mg (1 mmol) of MPI were ground with the addition of 10 μL of MeCN (Figure S5).

1.6 MPITFA

MPI (174.20 mg, 1 mmol)) and dilute triflic acid (0.5mmol, 500 μL) were added to 2 mL of MeCN and slowly evaporated under ambient conditions. Colorless block crystals of MPITFA were obtained within 10 days and were used for single-crystal X-ray crystallography. In SDG method,

324.27 mg (1 mmol) of 1-(4-methoxyphenyl)-1H-imidazolium triflate (salt8) and 174.20 mg (1 mmol) of MPI were ground with the addition of 10 μL of MeCN (Figure S6).

1.7 IMBHBA

IMB (172.18 mg, 1 mmol)) and dilute hydrobromic acid (0.5 mmol, 500 μL) were added to 2 mL of a 2:1 MeCN/MeOH solution and slowly evaporated under ambient conditions. Colorless block crystals of IMBHBA were obtained within 2 days. In the SDG method, 253.08 mg (1 mmol) of related salt (salt9) and 172.18 mg (1 mmol) of IMB were ground with the addition of 10 μL of MeCN (Figure S7).

1.8 NPIHBA

NPI (189.17 mg, 1 mmol) and dilute hydrobromic acid (0.5 mmol, 500 μL) were added to 2 mL of a 2:1 MeCN/MeOH solution and slowly evaporated under ambient conditions. The yellowish block crystals of **NPIHBA** were obtained within 10 days. In SDG method, 269.17 mg (1 mmol) of the related salt (salt12) and 189.17 mg (1 mmol) of NPI were ground with 10 μL of MeCN and a yellowish powder was produced (Figure S8).

1.9 NPIHCA

NPI (189.17mg, 1 mmol) and dilute hydrochloric acid (0.5 mmol, 500 μL) was added to 2 mL of a 2:1 MeCN/MeOH solution and slowly evaporated under ambient conditions. The yellowish block crystals of **NPIHCA** were obtained within 10 days (Figure S9).

1.10 NPINIA

NPI (0.0141 g, 0.13 mL) and dilute nitric acid (0.020 g, 0.13 μL) were dissolved in 2 mL of a 2:1 MeCN/MeOH solution. Slow evaporation of the solution afforded yellowish crystals of **NPINIA**. In SDG method, 225.57 mg (1 mmol) of 1-(4-nitrophenyl)-1H-imidazolium nitrate (salt14) and 189.17 mg (1 mmol) of NPI were ground with the addition of 10 μL of MeCN (Figure S10)

1.11 NPITFA

NPI (189.17 mg, 1 mmol)) and dilute triflic acid (0.5 mmol, 500 μL) were dissolved in 2 mL of a 2:1 MeCN/MeOH solution and slowly evaporated under ambient conditions. The yellowish block crystals of **NPITFA** were obtained within 7 days and were used for single-crystal X-ray crystallography. In the SDG method, 339.17 mg (1 mmol) of 1-(4-nitrophenyl)-1H-imidazolium triflate (salt16) and 189.17 mg (1 mmol) of NPI were ground with the addition of 10 μL of MeCN (Figure S11).

1.12 MPTHBA

MPT (1 mmol, 175.2 mg) and dilute hydrobromic acid (0.5 mmol, 500 μL) were dissolved in 2 mL of MeCN and slowly evaporated under ambient conditions. Colorless block crystals of **MPTHBA** were obtained within 8 days (Figure S12).

1.13 MPTHCA and MPTHCA·$2H_2O$

MPT (1 mmol, 175.2 mg) and dilute hydrochloric acid (0.5mmol, 500 μL) were dissolved in 2 mL of a 2:1 MeCN/MeOH solution and slowly evaporated under ambient conditions. Colorless block crystals of **MPTHCA** were obtained within 7 days. In the SDG method, 211.66 mg (1 mmol) of 1-(4-methoxyphenyl)-1H-1,2,4-triazolium chloride (salt18) and 175.19 mg (1 mmol) of MPT were ground in 10 μL of MeCN, resulting in the formation of the powder crystalline form of **MPTHCA** (Figure S13). Repeating this experiment with a 5:1 EtOH/H_2O ratio as the solvent results in the formation of **MPTHCA·$2H_2O$** (Figure S14).

1.14 MPTTFA

MPT (1 mmol, 175.19 mg) and dilute triflic acid (0.5 mmol, 500 μL) were dissolved in 2 mL of MeCN and slowly evaporated under ambient conditions. Colorless block crystals of **MPTTFA** were obtained within 10 days and were used for single-crystal X-ray crystallography. It is important to note the quality of the single crystal was poor. In the SDG method, 325.26 mg (1 mmol) of 1-(4-methoxyphenyl)-1H-1,2,4-triazolium triflate (salt21) and 175.19 mg (1 mmol) of MPT were ground with addition of 10 μL of MeCN (Figure S15).

1.15 MPIBMPT

(1-(4-methoxyphenyl)-1H-imidazole). (1-(4-methoxyphenyl)-1H-1,2,4-triazole). chloride, 17. To 210.66 mg (1 mmol) of 1-(4-methoxyphenyl)-1H-imidazolium chloride (salt5) was added 1 mmol of MPT which was then ground with the addition of 10 μL of MeOH. The obtained powder was used for recrystallization in 2 mL of MeOH under ambient conditions. Colorless needle-like single crystals of **MPIBMPT** were obtained within 6 days.

Section S2: Comparison of experimental and calculated PXRD patterns

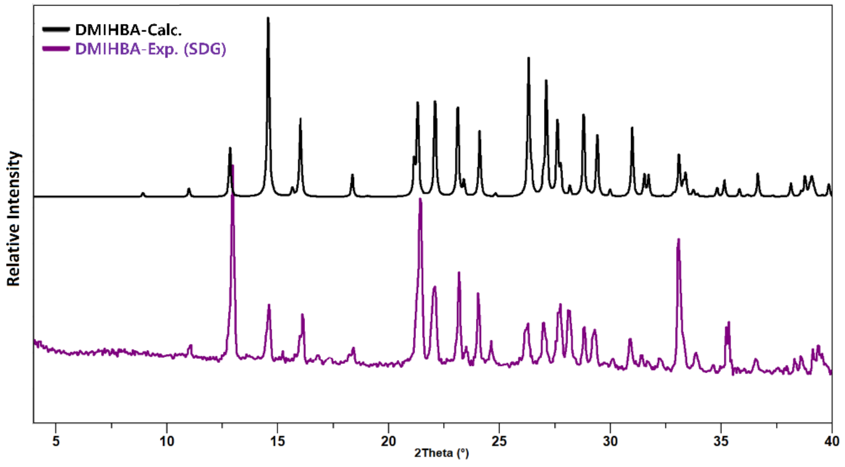

Figure S1. Experimental and calculated PXRD patterns of DMIHBA (DMI is liquid).

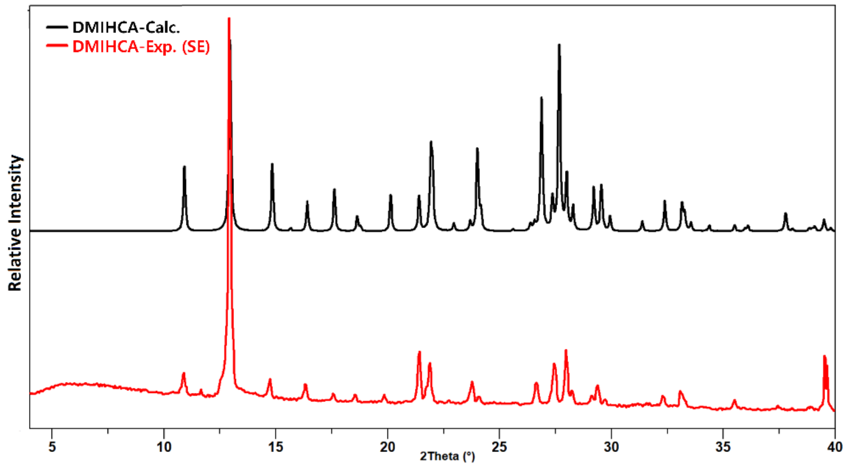

Figure S2. Experimental and calculated PXRD patterns of BEPTEX (DMI is liquid).

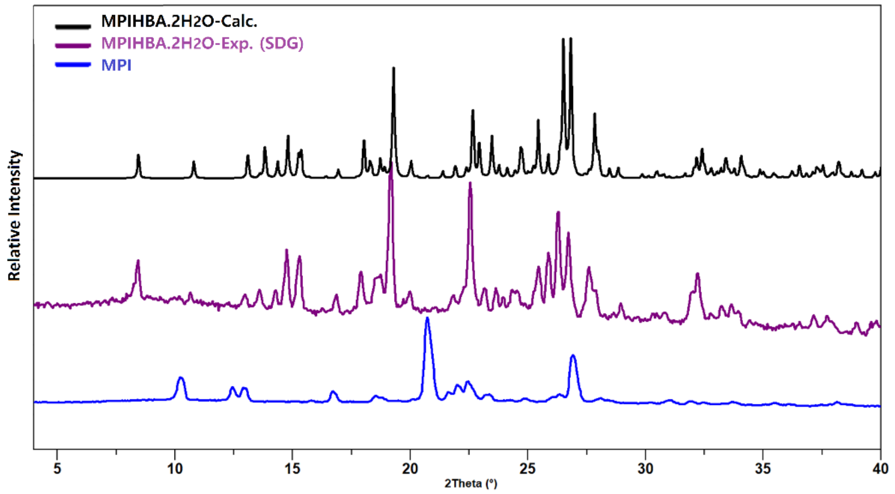

Figure S3. Experimental and calculated PXRD patterns of MPIHBA·$2H_2O$

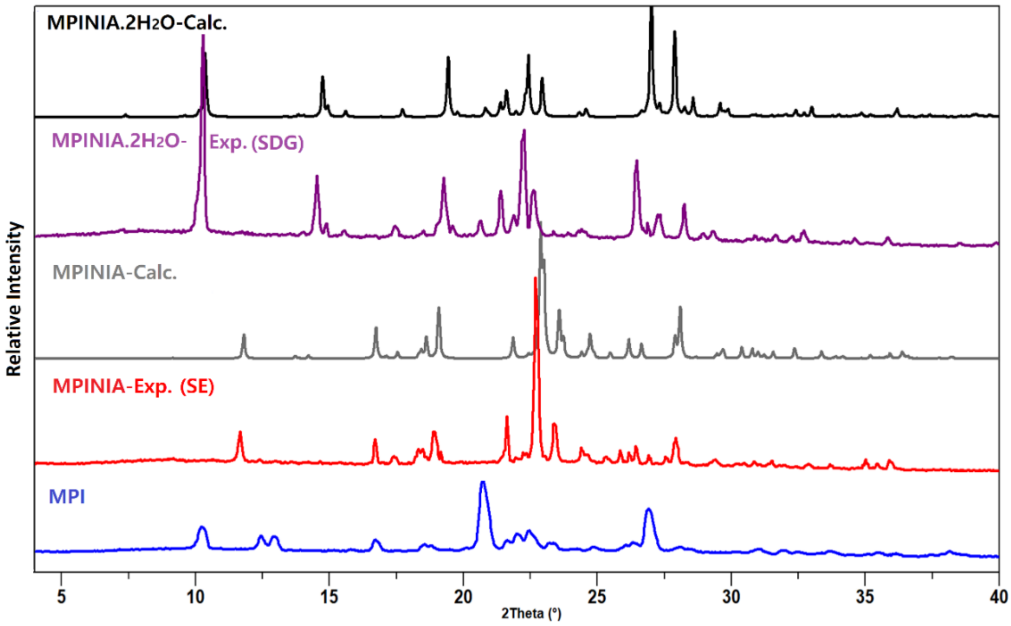

Figure S4. Experimental and calculated PXRD patterns of MPINIA and MPINIA.$2H_2O$.

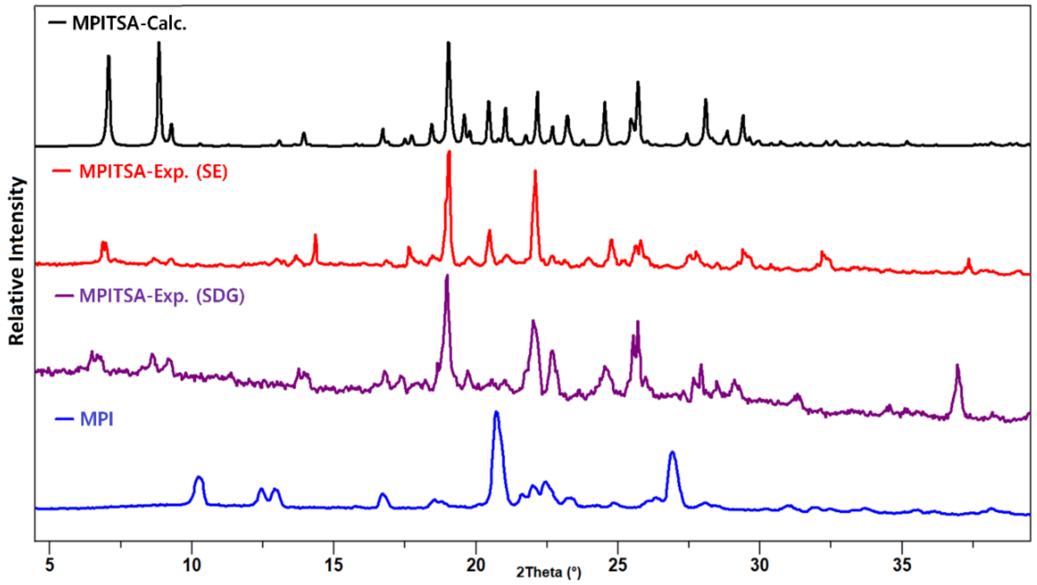

Figure S5. Experimental and calculated PXRD patterns of MPITSA.

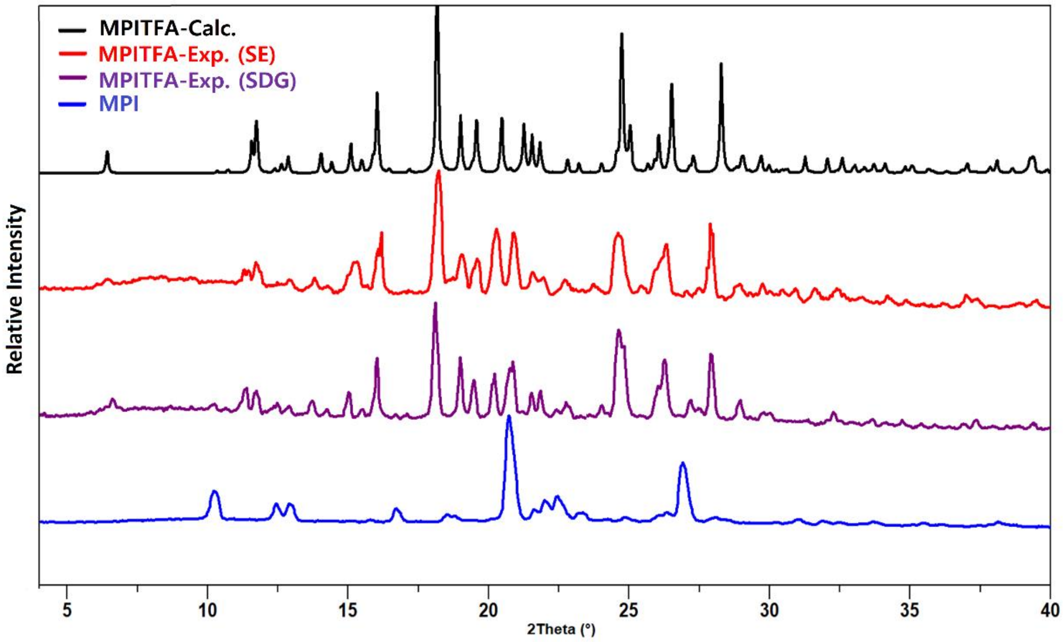

Figure S6. Experimental and calculated PXRD patterns of MPITFA.

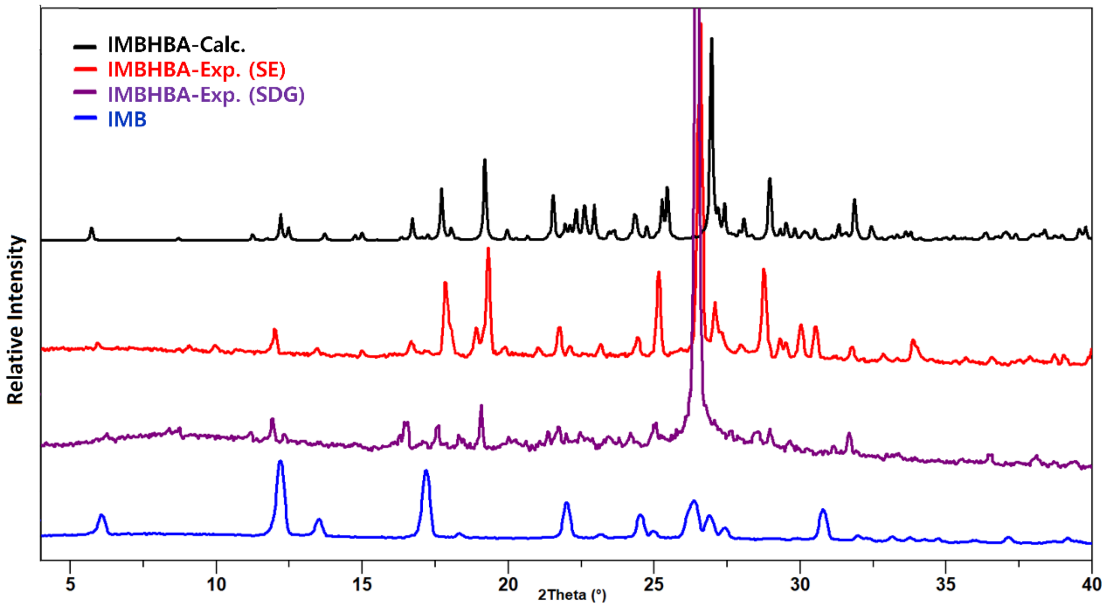

Figure S7. Experimental and calculated PXRD patterns of IMBHBA.

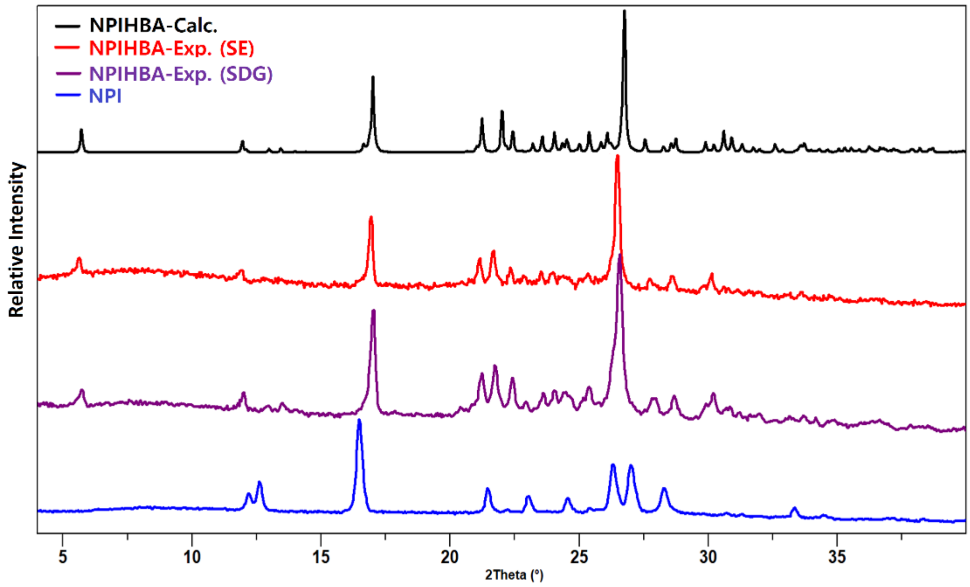

Figure S8. Experimental and calculated PXRD patterns of NPIHBA

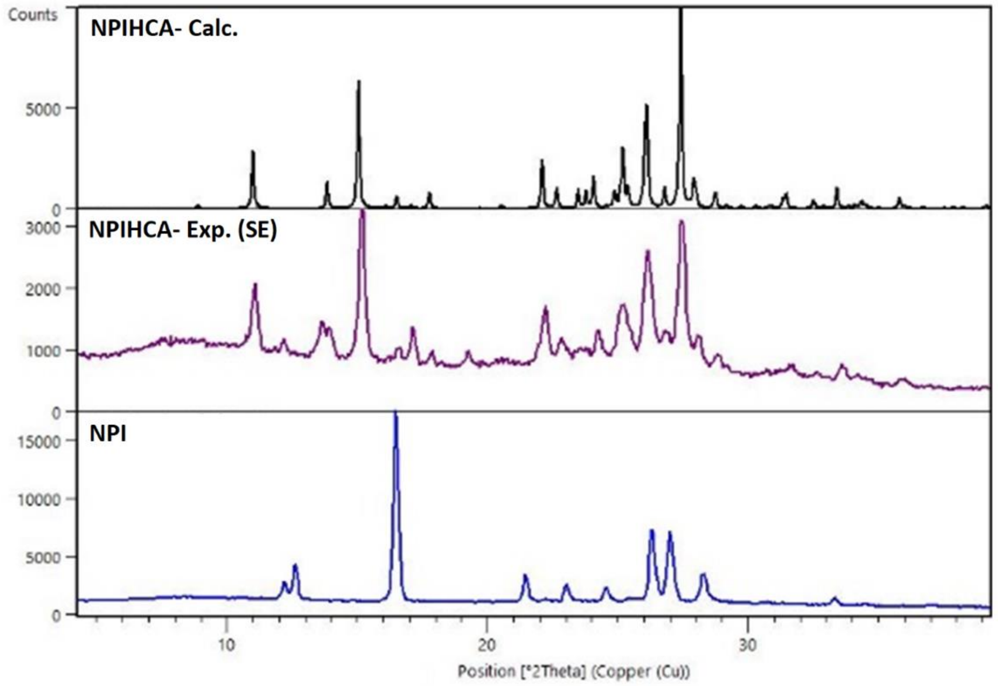

Figure S9. Experimental and calculated PXRD patterns of NPIHCA.

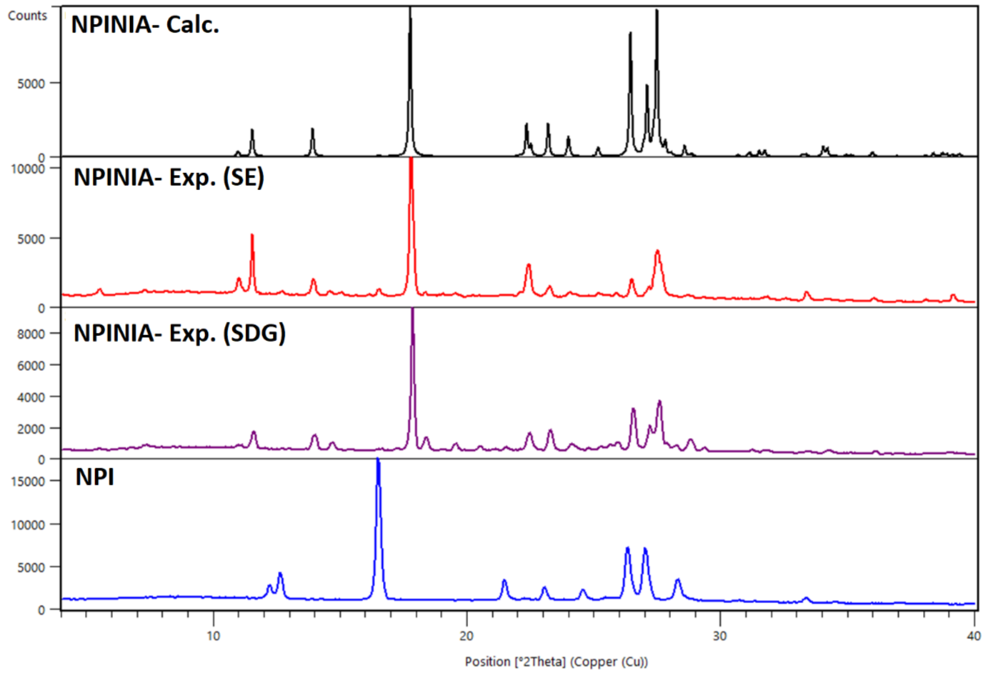

Figure S10. Experimental and calculated PXRD patterns of NPINIA.

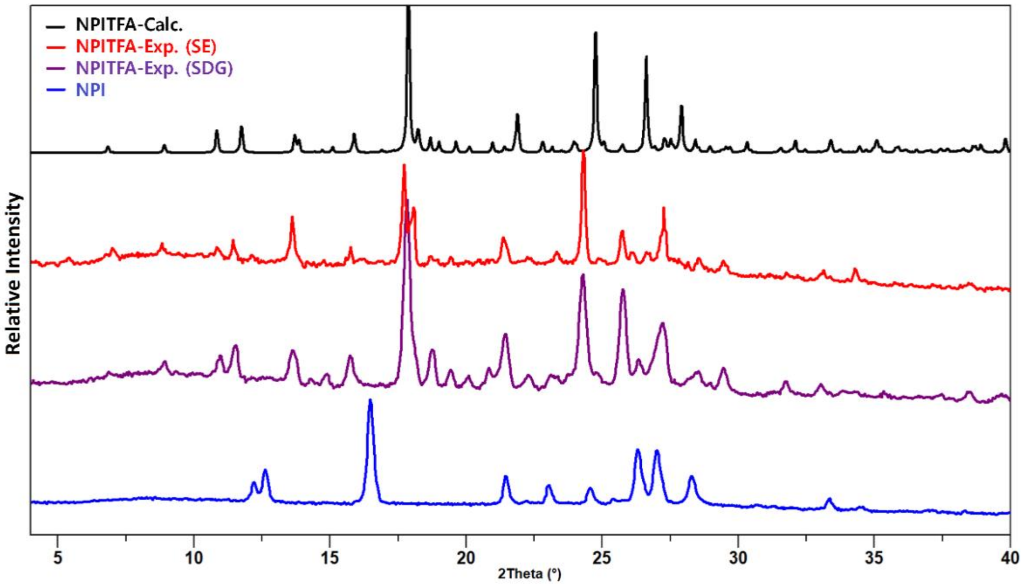

Figure S11. Experimental and calculated PXRD patterns of NPITFA.

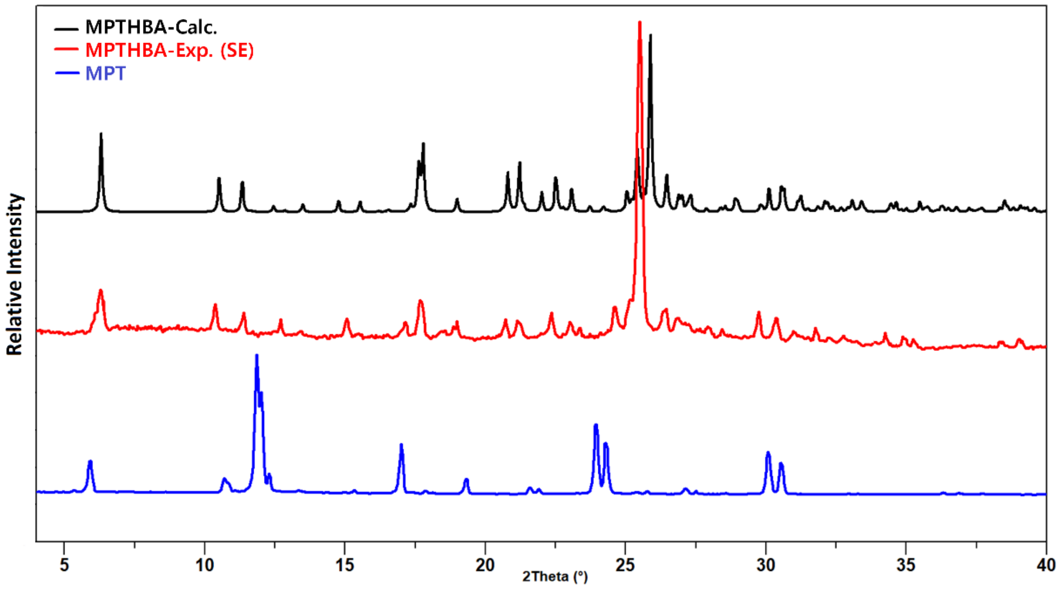

Figure S12. Experimental and calculated PXRD patterns of MPTHBA.

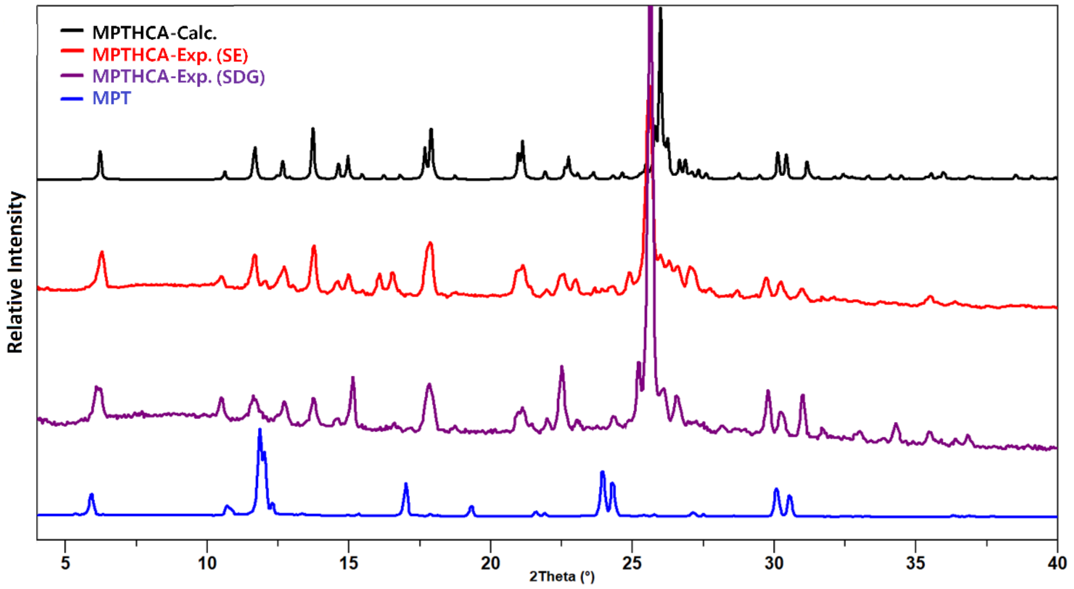

Figure S13. Experimental and calculated PXRD patterns of MPTHCA.

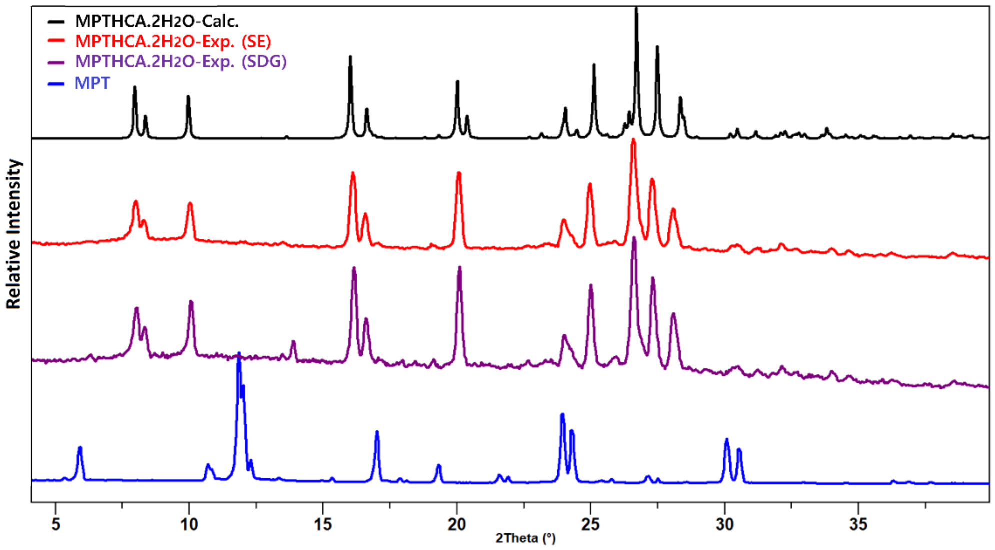

Figure S14. Experimental and calculated PXRD patterns of MPTHCA.2H_2O.

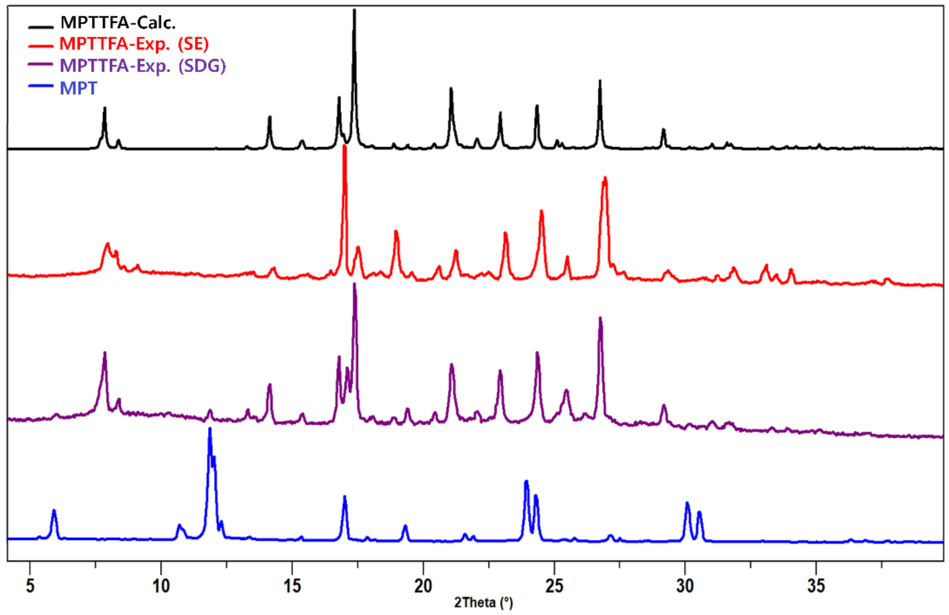

Figure S15. Experimental and calculated PXRD patterns of MPTTFA.

Section S3: Cambridge Structural Database (CSD) analysis.

ConQuest (v.5.42, Nov 2021) was used to search the Cambridge Structural Database, and Mercury was used to evaluate the results (2021.3.0). In all cases, the cut-off distance for the interatomic interactions between the two nitrogen atoms was set equal to the sum of vdW radii +0.3 Å. This precaution was justified by the possible errors associated with the position of hydrogen atom. We performed a CSD survey of azoles, which are chemical compounds having a five-membered aromatic ring structure and one or more heteroatoms, at least one of which must be a nitrogen atom. In this survey, aromatic nitrogen moieties containing at least one extra nitrogen, oxygen, or sulfur atom as part of the ring were undertaken, which may act as azole rings (A or C). The search for charge-assisted supramolecular heterosynthons between two identical or distinct azole rings using CSD can reveal how many azole-based structures include the heterosynthons and parameters including interaction distance and angle of each ring. For this search, we established the following parameters: only single crystal structures, 3D coordinates, and R<10%. Contact limits for each interaction were determined was set equal to the sum of vdW radii +0.3 Å for selected entries. The N atom in the neutral ring was specified as uncharged, and the number of bonded atoms was set to two. With the presence of the hydrogen atom bound to the nitrogen, the charge of the nitrogen was set to +1 and the number of bonded atoms was set to 3.

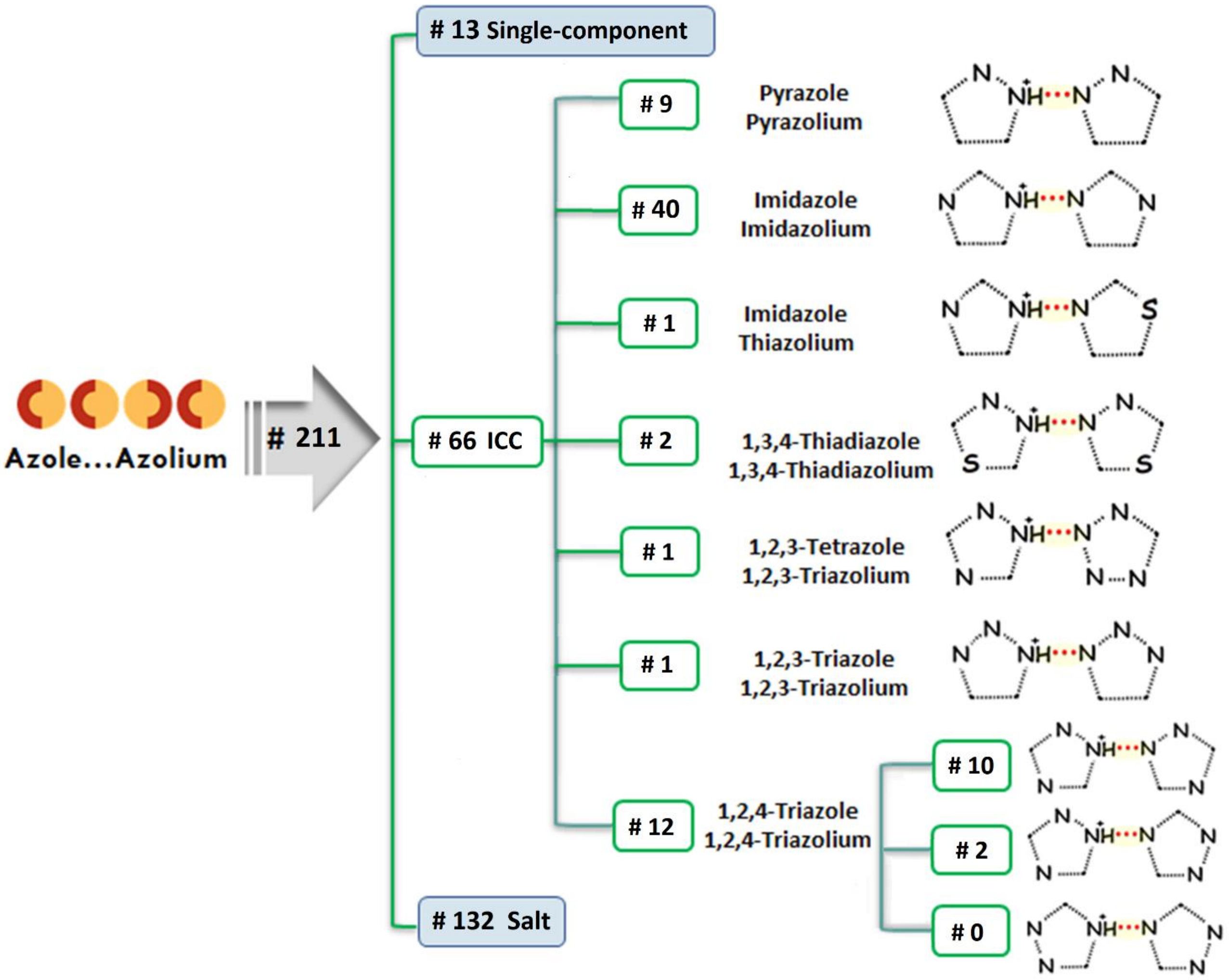

Scheme S1. CSD statistics for supramolecular synthons in structures containing azole rings.

Table S3. CSD statistics for supramolecular synthons sound in structures containing azole rings.

Refcode	Type	N···N/Å	α/°	θ_1/°	θ_2/°	Refcode	Type	N···N/Å	α/°	θ_1/°	θ_2/°
**AHOJUD**	A^+B^-C	2.915	156.42	103.77	102.74	**NILQEF**	A^+B^-A	2.653	153.71	107.67	111.24
**ABIZES**	A^+B^-A	2.721	172.38	106.03	107.71	**NOLDEA**	A^+B^-A	2.725	165.26	106.14	108.82
**AHILEL**	A^+B^-A	2.765	172.013	110.03	112.19	**NOLVIV**	A^+B^-A	2.921	168.56	104.53	112.20
**BAXSIF**	A^+B^-A	2.675	170.92	109.33	109.26	**OKACEK**	A^+B^-A	2.685	173.80	106.89	110.26
**BEPTEX**	A^+B^-A	2.711	174.92	105.99	109.13	**ONAWOQ**	A^+B^-A	2.688	162.96	105.64	108.67
**BOZPOY**	A^+B^-C	2.746	164.15	104.48	109.15	**OPIKAA**	A^+B^-A	2.695	151.40	106.73	107.51
**BOZPUE**	A^+B^-C	2.741	165.88	104.64	108.45	**OYUZEO**	A^+B^-A	2.747	166.54	107.42	111.33
**BUQQEK**	A^+B^-A	2.739	173.36	105.73	105.83	**PEKXIO**	A^+B^-A	2.811	166.22	105.31	110.14
**BUQRAH**	A^+B^-A	2.686	177.36	104.72	107.47	**QAKGUG**	A^+B^-A	2.665	179.62	106.09	106.64
**CIBDID**	A^+B^-A	2.683	149.80	102.66	110.81	**RUZHEC**	A^+B^-A	2.652	160.21	108.95	109.87
**DUFBAJ**	A^+B^-A	2.686	165.17	107.06	111.03	**RIDKIZ**	A^+B^-A	2.696	153.87	107.51	111.24
**DUVZEC**	A^+B^-A	2.705	175.79	106.17	108.94	**RUDRIR**	A^+B^-A	2.794	134.91	106.06	109.91
**DUXJUD**	A^+B^-A	2.745	170.76	104.29	107.10	**SERHOP**	A^+B^-C	2.715	154.55	105.06	108.89
**EGUANC**	A^+B^-A	2.636	175.58	105.97	106.44	**TABDIK**	A^+B^-A	2.772	164.66	108.94	114.86
**ENINIA**	A^+B^-A	2.663	173.68	106.22	106.95	**TABDOQ**	A^+B^-A	2.828	171.17	105.19	112.37
**ENUHAX**	A^+B^-A	2.679	172.86	105.95	107.71	**TABDUW**	A^+B^-A	2.785	164.89	102.03	99.67
**ERAHIO**	A^+B^-A	2.867	166.71	107.75	119.23	**TAZYAX**	A^+B^-C	2.761	157.91	103.91	111.26
**FIXLII**	A^+B^-A	2.739	175.56	105.68	106.71	**TIHDIX**	A^+B^-A	2.689	174.99	108.13	111.05
**FOHKIX**	A^+B^-A	2.686	177.53	106.83	107.44	**TUTMED**	A^+B^-C	2.840	162.69	111.01	111.97
**GAGKOS**	A^+B^-A	2.753	169.89	104.72	108.09	**UMAHOH**	A^+B^-A	2.728	162.97	105.80	108.75
**GEHFIL**	A^+B^-A	2.710	170.40	110.57	111.30	**VOQFOZ**	A^+B^-C	2.775	160.42	104.65	109.00
**GUFKOH**	A^+B^-A	2.714	168.76	106.16	107.98	**WOGHOR**	A^+B^-A	2.741	163.72	111.04	115.16
**HATQIF**	A^+B^-C	2.821	167.94	107.42	105.69	**WOSSOP**	A^+B^-A	2.717	157.41	103.79	113.64
**HUMMOS**	A^+B^-A	2.688	174.79	106.07	108.62	**WUGRUO**	A^+B^-A	2.736	166.45	107.54	106.60
**HUMMUY**	A^+B^-A	2.739	171.16	106.51	109.39	**XAZVOK**	A^+B^-A	2.872	176.16	103.38	107.65
**IGUZIW**	A^+B^-A	2.790	168.64	105.24	110.15	**XIVZIN**	A^+B^-A	2.759	161.86	113.54	117.08
**IJIBEI**	A^+B^-A	2.694	174.62	108.00	112.18	**XUPLEZ**	A^+B^-A	2.843	144.57	107.19	104.67
**KAMBIK**	A^+B^-A	2.802	143.50	107.58	105.64	**YAWYIG**	A^+B^-A	2.718	175.13	106.41	106.41
**LEZSAL**	A^+B^-A	2.729	179.75	106.35	107.95	**YOMSUP**	A^+B^-A	2.821	171.47	105.32	109.33
**LIPROR**	A^+B^-A	2.700	173.13	106.62	107.38	**ZIDJUT**	A^+B^-A	2.735	172.86	104.85	105.96
**LUMKOV**	A^+B^-A	2.727	164.81	107.59	111.82	**KABJIH**	A^+B^-C	2.786	174.50	104.67	109.11
**MEHKEQ**	A^+B^-A	2.703	167.64	105.73	106.87	**OQIHEE**	A^+B^-A	2.671	174.49	108.05	109.19
**MOYYOO**	A^+B^-A	2.660	174.30	107.14	106.50	**REYMOX**	A^+B^-C	2.881	169.60	110.96	108.81

Section S4. Crystal structure analysis.

In S4 we have depicted cations in blue, anions in red, and neutral molecules in green.

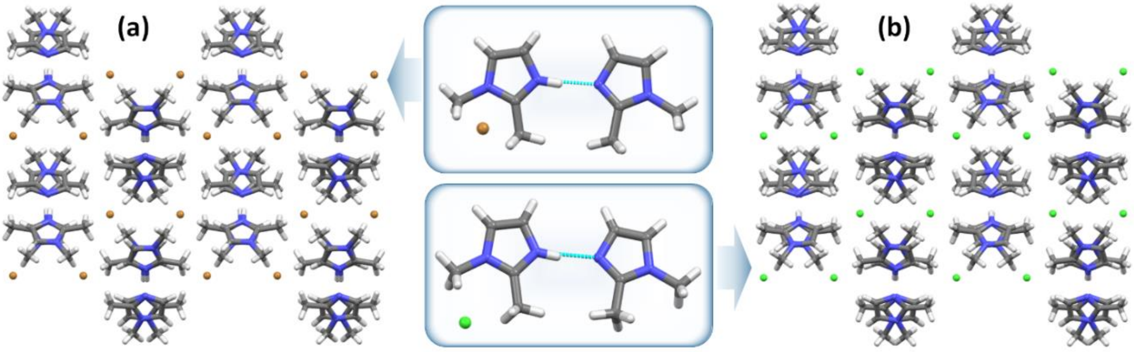

Figure S16. Illustration of isostructural ICCs DMIHBA (a) and BEPTEX (b) along *c* axis.

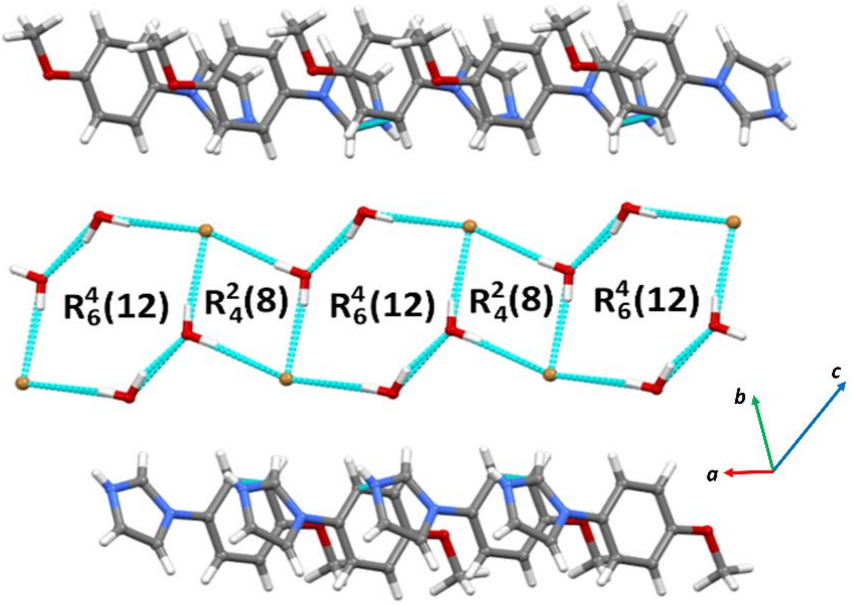

Figure S17. Graph-set analysis of water molecules in MPIHBA·2H2O

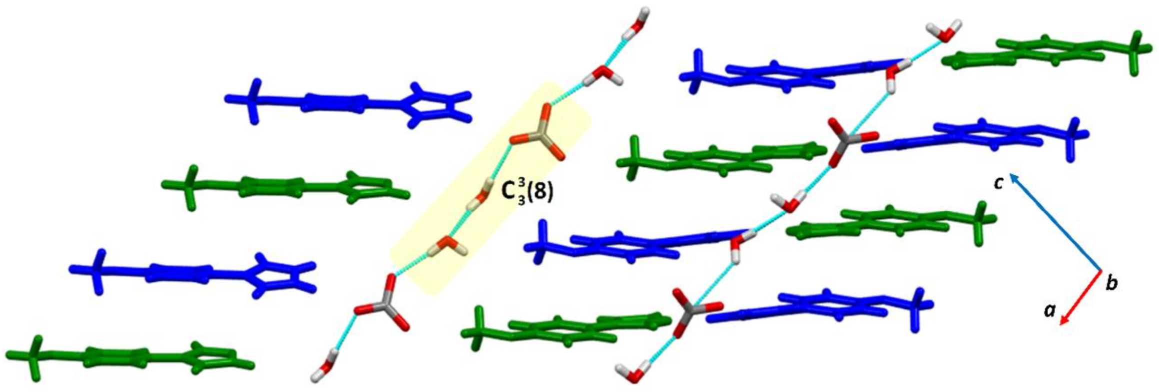

Figure S18. The chain arrangement of nitrate anions and water molecules in MPINIA·2H₂O.

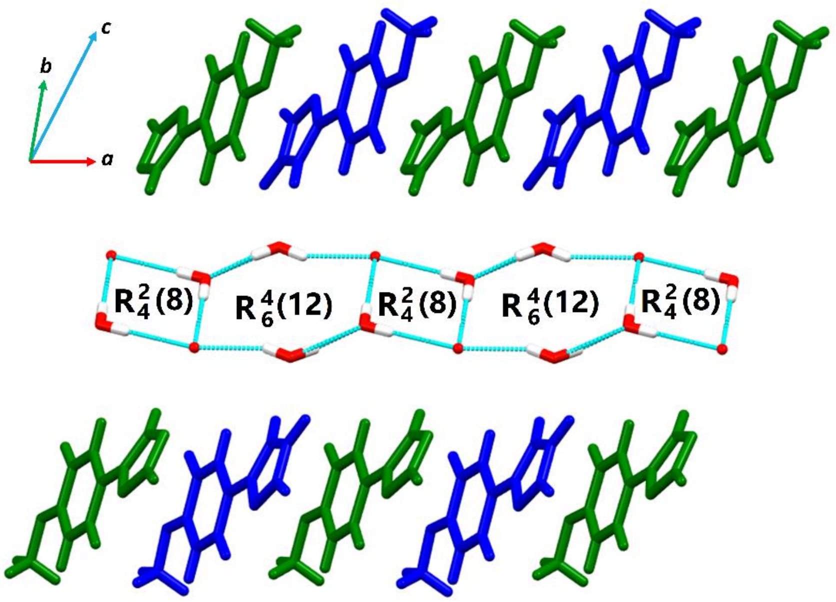

Figure S19. Graph-set analysis of water molecules in MPTHCA·2H₂O.

Section S5. Hydrogen bond analysis.

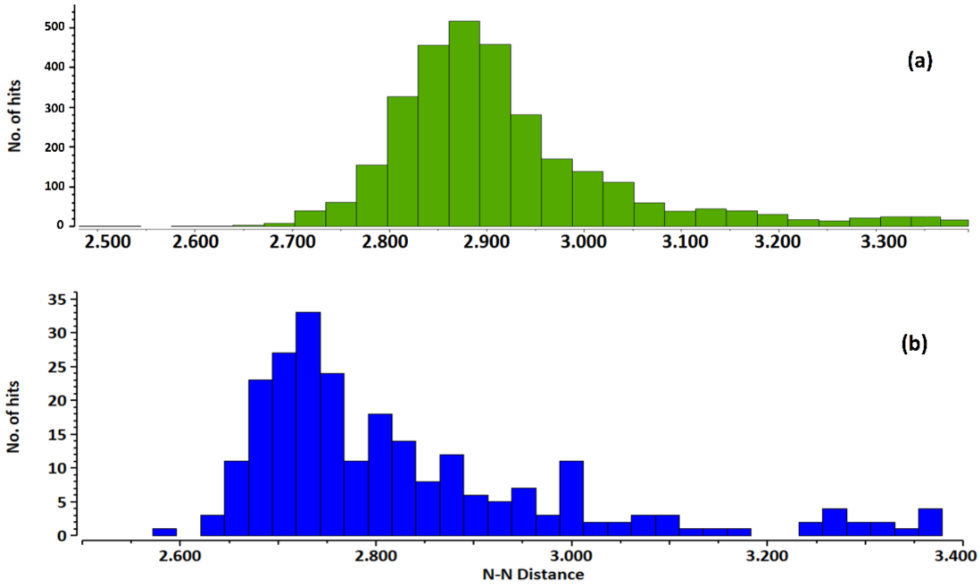

Figure S20. CSD statistics for azole-ring structures are plotted for 2129 neutral NH···N hydrogen bonds with the average bond length of 2.890 ± 0.032 Å (green) (a) and 211 charge-assisted NH^+···N synthons with the average bond length of 2.740 ± 0.027 Å (blue) (b).
